# Use of a Novel Deep Learning Open-Source Model for Quantification of Ki-67 in Breast Cancer Patients in Pakistan: A Comparative Study between the Manual and Automated Methods

**DOI:** 10.3390/diagnostics13193105

**Published:** 2023-09-30

**Authors:** Talat Zehra, Nazish Jaffar, Mahin Shams, Qurratulain Chundriger, Arsalan Ahmed, Fariha Anum, Najah Alsubaie, Zubair Ahmad

**Affiliations:** 1 Department of Pathology, Jinnah Sindh Medical University, Karachi 75001, Pakistan; talat.zehra@jsmu.edu.pk (T.Z.); nazish.jaffer@jsmu.edu.pk (N.J.); 2 Department of Pathology, United Medical and Dental College, Karachi 71500, Pakistan; mahin.16@gmail.com; 3 Department of Pathology and Laboratory Medicine, Section of Histopathology, Aga Khan University Hospital, Karachi 3500, Pakistan; chundriger_86@hotmail.com (Q.C.); arsalan.ahmed@aku.edu (A.A.); 4 Research Department, Ziauddin University, Karachi 75600, Pakistan; fariha.anum@zu.edu.pk; 5 Department of Computer Sciences, College of Computer and Information Sciences, Princess Nourah bint Abdulrahman University (PNU), P.O. Box 84428, Riyadh 11671, Saudi Arabia; 6 Consultant Histopathologist, Sultan Qaboos Comprehensive Cancer Care and Research Centre, Seeb P.O. Box 556, Oman; z.ahmad@cccrc.gov.com

**Keywords:** AI (artificial intelligence), breast neoplasms, deep learning, computer-assisted image processing, immunohistochemistry, Ki-67 antigen

## Abstract

**Introduction:** Breast cancer is the most common cancer in women; its early detection plays a crucial role in improving patient outcomes. Ki-67 is a biomarker commonly used for evaluating the proliferation of cancer cells in breast cancer patients. The quantification of Ki-67 has traditionally been performed by pathologists through a manual examination of tissue samples, which can be time-consuming and subject to inter- and intra-observer variability. In this study, we used a novel deep learning model to quantify Ki-67 in breast cancer in digital images prepared by a microscope-attached camera. **Objective:** To compare the automated detection of Ki-67 with the manual eyeball/hotspot method. Place and duration of study: This descriptive, cross-sectional study was conducted at the Jinnah Sindh Medical University. Glass slides of diagnosed cases of breast cancer were obtained from the Aga Khan University Hospital after receiving ethical approval. The duration of the study was one month. **Methodology:** We prepared 140 digital images stained with the Ki-67 antibody using a microscope-attached camera at 10×. An expert pathologist (P1) evaluated the Ki-67 index of the hotspot fields using the eyeball method. The images were uploaded to the DeepLiif software to detect the exact percentage of Ki-67 positive cells. SPSS version 24 was used for data analysis. Diagnostic accuracy was also calculated by other pathologists (P2, P3) and by AI using a Ki-67 cut-off score of 20 and taking P1 as the gold standard. **Results:** The manual and automated scoring methods showed a strong positive correlation as the kappa coefficient was significant. The *p* value was <0.001. The highest diagnostic accuracy, i.e., 95%, taking P1 as gold standard, was found for AI, compared to pathologists P2 and P3. **Conclusions:** Use of quantification-based deep learning models can make the work of pathologists easier and more reproducible. Our study is one of the earliest studies in this field. More studies with larger sample sizes are needed in future to develop a cohort.

## 1. Introduction

Breast cancer is the leading cause of cancer-related deaths in women worldwide. The early detection and accurate evaluation of the proliferation of cancer cells play a crucial role in the management of this disease. Ki-67 is a well-established biomarker used in the assessment of breast cancer patients, as it provides valuable information on the rate of cell division and the prognosis of the disease [1]. Ki-67 is also referred to as the marker of proliferation and is encoded by the MKI67 gene. Since Ki-67 remains active during the G1, S, G2, and M phases of the cell cycle, it is a very accurate indicator of cell proliferation and a widely recognised indicator of oncogenesis. Due to its association with the proliferative activity of cancer cells, the immunohistochemical examination of Ki-67 is currently included in the paradigm for a variety of tumour types. In a range of malignancies, including breast tumours, neuroendocrine tumours, and gastrointestinal stromal tumours (GIST), reliable analysis utilizing Ki-67 as the sole biomarker has been verified [2]. Therefore, it could be utilised to estimate outcomes over time and, in certain instances, to predict responsiveness to specific treatments like chemotherapy and endocrine therapy [1].

A large number of luminal tumours, which account for 70% of all occurrences of breast cancer, are hormone receptor (HR)-positive. Endocrine therapy is extremely useful for luminal A cancers, which have a low proliferation and a better prognosis, while luminal B tumours, which have a significant proliferation and a worse prognosis, are less sensitive to it. HER2-enriched subtypes are aggressive tumours with a poor prognosis. Ki-67 is crucial for identifying luminal A-like and luminal B-like tumours among HR+/HER2 malignancies, and by doing so, determining the need for chemotherapy. Over the past three decades, numerous efforts have been made to assess the Ki-67 proliferation index’s prognostic potential. However, the inability to standardise Ki-67 assessment methods has prevented this biomarker from becoming a fully integrated part of clinical decision making or pathological reporting [3]. Traditionally, Ki-67 quantification has been performed through a manual examination of tissue samples by pathologists. This method is time-consuming and subjective, leading to inter- and intra-observer variability. Furthermore, manual examination is limited by the expertise of the observer and the results can be impacted by factors such as fatigue and eyestrain [1,4].

The Ki-67 biomarker needs a more precise and well-defined scoring system because it has not yet been widely standardised, unlike other immunohistochemistry markers like the oestrogen receptor, progesterone receptor, and HER2. This has restricted its use in both research and diagnostic contexts [1]. Inconsistencies in scoring are inevitable, according to the International Ki-67 Working Group’s (IKWG) recommendations. Differences in Ki-67 scores can result from the type of specimen, such as cytological or histological, and from individual pathologists’ observational differences [5]. In addition, there are several techniques used for staining and scoring Ki-67, which may result in scoring discrepancies [2,5]. Therefore, the IKWG has suggested the development of an automated Ki-67 scoring system to overcome these limitations to the optimal utilization of this marker [5].

Recently, advances in artificial intelligence and computer vision have led to the development of automated methods for Ki-67 quantification. These methods use deep learning algorithms to analyse images of tissue samples and accurately quantify the level of Ki-67 expression. The use of these algorithms has the potential to significantly improve the accuracy, speed, and consistency of Ki-67 quantification [6,7]. Various studies have been conducted on the automated scoring of the Ki-67 index [8]. Boyaci et al. assessed its reproducibility among pathologists utilizing artificial intelligence algorithms. By reaching intraclass correlation coefficient values similar to those in the IKWG study, they proved that the artificial intelligence-based automated Ki-67 scoring method may be used to achieve good reproducibility compared to pathologists [9,10]. Furthermore, a comparative analysis of the visual assessment and automated digital image analysis of the Ki-67 index in breast cancer was performed by Zhong et al. [11] and found a significant degree of consistency between both methods [11].

In this study, we present concordance of manual and automated methods for Ki-67 quantification in breast cancer through an open-source software in patients belonging to South Asian regions. Our study aimed to evaluate the accuracy and performance of the automated method using annotated images. We also compared the results obtained by the novel deep learning model for Ki-67 quantification with the manual method. Furthermore, the current study also experimented with digital images captured via microscope-attached camera, in the absence of digital scanners, setting the stage for a low resource setup, where pathologists can benefit from AI-based deep learning models in a similar manner.

The results of this study will provide valuable insights into the potential use of automated methods for Ki-67 quantification and will demonstrate the advantages as well as limitations of both manual and automated methods. The findings of this study will have important implications for the clinical management of breast cancer patients and will contribute to the advancement of the field of medical image analysis.

## 2. Methodology

About 140 digital images of invasive ductal carcinoma of the breast stained with Ki-67 Immune marker were obtained from the Section of Histopathology, Department of Pathology and Laboratory Medicine, Aga Khan University Hospital after obtaining ethical approval from the Ethical Review Committee. The current study was carried out during a one-month period from 25 December 2022 to 25 January 2023.

**Figure 1:**
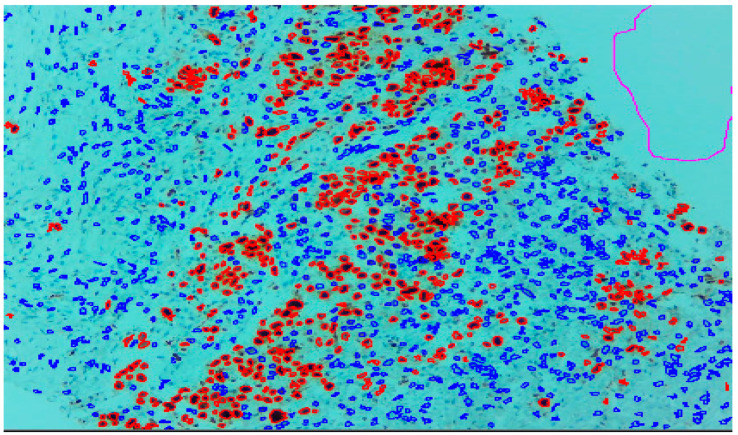
Ki-67 image at 10× showing positive tumour cells as red outlines and tumour negative cells as blue outline.

**Figure 2:**
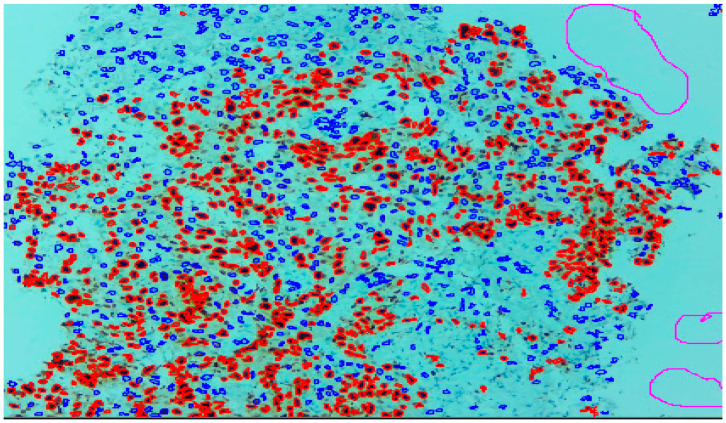
With the help of tools regions where software has picked up wrongly can be excluded with the exclude region tool, used here as magenta outlines in both Figure 1 and Figure 2.

**Figure 3:**
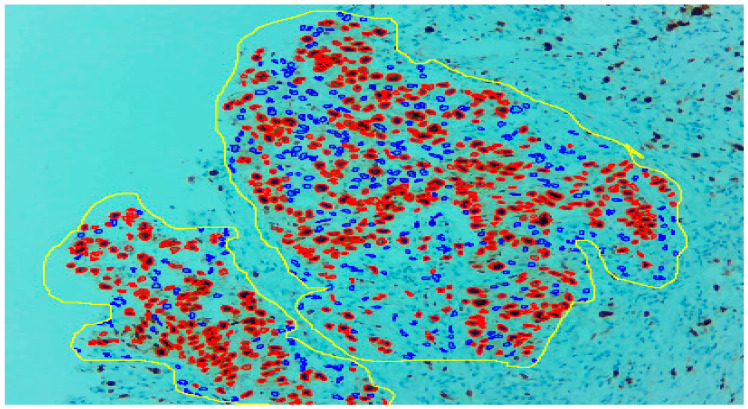
With the help of the excluding region tool as yellow outline, only the tumour region can be selected.

Three expert pathologists evaluated the Ki67 index in the hotspot fields using the manual eyeball method. The average number of cells was around 1400 in each specimen. Digital images were taken from the hotspot areas of tumour and all slides were digitalized at 10×. The score of the pathologist P1 was considered as the gold standard for those who manually quantified both tumour positive and negative cells. The images were uploaded to the open-source DeepLiif software [12,13]. This particular software helped to detect and quantify Ki67 positive and negative tumour cells along with the percentage of tumour positive cells. The cells which were positive for Ki-67 showed red outlines while tumour negative cells showed blue outlines (Figure 1). We used different tools available in the software including size gating, marker threshold as well as excluding the regions for achieving more accurate and reproducible results (Figure 2 and Figure 3). DeepLIIF provides an exclusion/inclusion region-of-interest lasso/selection tool that was used to exclude all the stromal cells after running, as shown in Figure 3. Scores prepared by the remaining two pathologists (P2 and P3) and scores provided by AI-based software were compared with the scores of pathologist P1. 

DeepLIIF software is a state-of-the-art tool for clinical IHC Ki-67 quantification. It uses a novel approach for virtual/digital multiplex immunofluorescence re-staining of clinical IHC slides to outperform other previous state-of-the-art algorithms. More information on the rigorous benchmarking and comparisons with state-of-the-art algorithms can be found in the three cited Ghahremani et al., *Nature Machine Intelligence* 2022 [12], CVPR 2022 [13], and MICCAI 2023 [14] papers.

DeepLIIF [12,13,14] uses a multitask-supervised deep learning approach to digitally/virtually re-stain clinical IHC slides with multiplex immunofluorescence staining while simultaneously performing semantic segmentation to differentiate between IHC+/− cells. Specifically, DeepLIIF represents a novel supervised generative adversarial network approach for virtual re-staining of clinical slides.

DeepLIIF is also a completely open-source platform with code, pretrained AI models, and training/testing datasets publicly available for reproducibility and full transparency. It is also the only AI IHC scoring model available for free via cloud-native platform with user-friendly interface (https://deepliif.org, accessed on 25 December 2022) for anyone in the world to upload their images and obtain results including developing region pathologists facing financial constraints in being able to afford digital scanners. DeepLIIF supports both scanned images as well as microscope snapshots (with large tumour coverage at 10×); no commercial solutions support uploading of microscope snapshots at 10× and only support 20× or 40× which has a low coverage for developing regions. This makes DeepLIIF the only advanced AI solution currently available to low/limited resource developing region settings that need it the most.

### Statistical Analysis

The data were entered and analysed by using SPSS Version 24. Normality of continuous data was assessed by using the Kolmogorov–Smirnov test. The data were represented by Med [Q_1_, Q_3_]. Agreement between P1 with P2, P3, and AI was measured by applying Kappa analysis and validity was assessed by correlation. Cronbach’s alpha and intraclass correlation coefficient (ICC) were also calculated to assess the internal consistency and reliability, respectively. ROC was plotted and AUC was evaluated for each variable to find the quality of test. Diagnostic accuracy was also calculated for P2, P3, and AI by using Ki67 cut-off score as 20 and taking P1 as gold standard. The Bland–Altman analysis was also performed to visualize the agreement of P2, P3, and AI with the reference standard P1, for the quantification of the Ki67 score.

## 3. Results

By using the Kolmogorov–Smirnov test, the distribution of P1, P2, P3, and AI were found to be non-normal (*p*-value < 0.05). The median [Q_1_, Q_3_] for P1 is 15 [10, 24], P2 is 15 [10, 22], P3 is 20 [10, 25] and for AI is 14.15 [10, 25], as reported in Table 1.

In Table 2, the agreement measure of P1 was assessed with P2, P3, and AI to observe the inter-rater reliability. Out of 140 cases, evaluated by P1 and P2, 92 cases have Ki-67 score ≤ 20, as agreed by both pathologists (P1 and P2). In addition, both pathologists agreed that there were 29 cases which have Ki-67 score > 20. Therefore, there were 19 cases (i.e., 8 + 11 = 19) for whom the two pathologists could not agree. So, statistically the value of kappa was found to be 0.660, which indicates a good strength of agreement.

Similarly, these 140 cases were also evaluated by P1 and P3. Out of which, 88 cases have a Ki-67 score ≤ 20 that was agreed by both pathologists (P1 and P3). Also, they agreed that 36 cases have a Ki-67 score > 20. But there were 16 (i.e., 12 + 4 = 16) such cases for which these two pathologists could not agree. The Kappa statistic was found to be 0.736 that shows a good strength of agreement.

Most importantly, these 140 cases were also evaluated by AI. The results showed that out of 140 cases, the pathologist P1 and AI were agreed that there are 94 cases that have a Ki-67 score ≤ 20 and 39 cases have a Ki-67 score > 20, while for 7 cases (i.e., 6 + 1 = 7) there was a disagreement between the pathologist P1 and AI. The Kappa statistic was calculated as 0.882 that indicates a very good strength of agreement between the pathologist P1 and AI.

Table 3 depicts the findings that were evaluated by ROC curves and area under the ROC curve (AUROC) through which we were able to assess the test quality and the best cut-off value by using Youden’s Index Method. The value of AUC for P2 was found to be 0.940 which indicates an excellent test quality with the best cut-off score for Ki-67 as 16.5, with TPR as 92.5%, and FPR as 20%.

The AUC value for P3 resulted in being 0.934, which also indicates the excellent quality of test with best cut-off score for Ki-67 as 24.5 with TPR as 90% and FPR as 11%.

Similarly, for AI, the value of AUC was found to be the highest as 0.993, indicating that the quality of the test is excellent. The best cut-off score for Ki-67 was found to be 21.5, with TPR as 97.5%, and FPR as 2%.

Table 4 was constructed to evaluate the diagnostic accuracy of P2, P3, and AI by using the Ki-67 cut-off score as 20, taking P1 as the gold standard. The sensitivity, specificity, PPV, NPV and diagnostic accuracy of P2 were calculated as 92%, 72.5%, 89.32%, 78.38%, and 86.43%, respectively.

Similarly, the sensitivity, specificity, PPV, NPV, and diagnostic accuracy of P3 were found to be 88%, 90%, 95.65%, 75%, and 88.57%, respectively.

For AI, the sensitivity, specificity, PPV, NPV and diagnostic accuracy were calculated as 94%, 97.5%, 98.95%, 86.67%, and 95%, respectively. As a whole, the highest diagnostic accuracy was found for AI, i.e., 95%, in comparison to other pathologists, by taking P1 as the gold standard.

**Figure 4:**
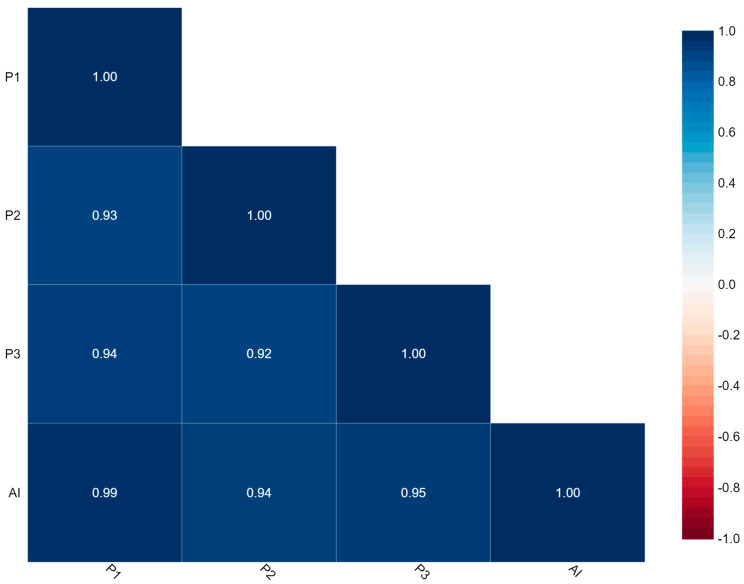
Correlation matrix.

Figure 4 represents the correlation between P1, P2, P3, and AI. The findings of Ki-67 by this correlation matrix showed that P1 is very strongly correlated with P2, P3, and AI with values of 0.93, 0.94, and 0.99, respectively.

Here, Cronbach’s alpha = 0.985 which indicates excellent internal consistency and Intraclass Correlation Coefficient (ICC) = 0.930 with 95% CI (0.900–0.951) which indicates excellent reliability.

**Figure 5:**
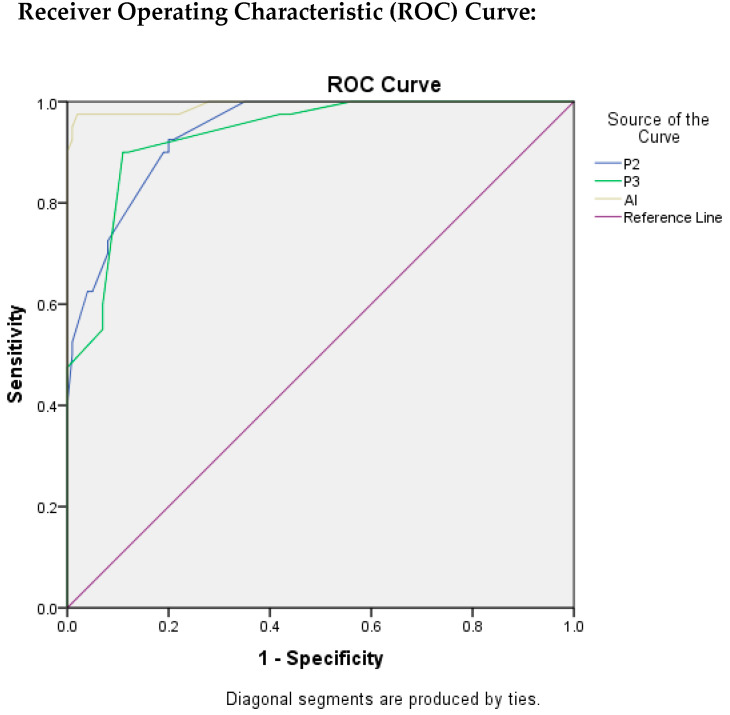
ROC Plots for P2, P3, and AI.

In Figure 5, ROC curves were plotted for P2, P3, and AI to evaluate the quality of the test, taking P1 as reference standard. 

**Figure 6:**
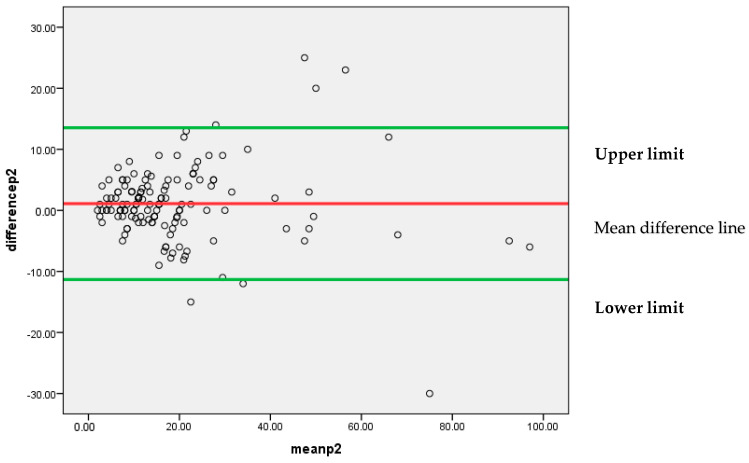
Bland–Altman plot for P1 and P2.

Figure 6 represents that most of the differences between the two pathologists’, P1 and P2, findings are lying between 95% confidence limits of agreement (−11.3307, 13.5349). Each individual data point on the plot represents the difference between the measurements for each Ki-67 score. The vertical position of each point indicates the difference between the two pathologists’ findings, while the horizontal position indicates the average of the two measurements. The scattered points around the line of mean difference provides insight into the variability of the differences. Since the majority of points are clustered around the mean difference line without a clear pattern, it suggests an insignificant systematic bias between the two pathologists’ findings. A narrow spread of the scattered points indicates low variability in the differences.

**Figure 7:**
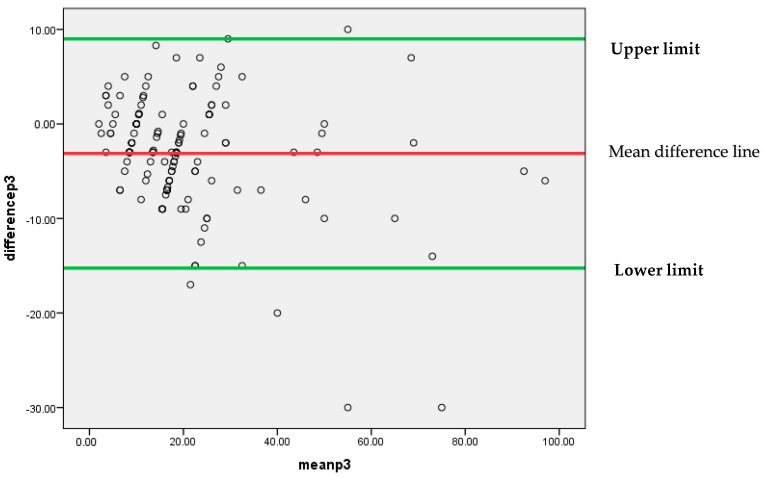
Bland–Altman plot for P1 and P3.

Similar to the above findings, Figure 7 also depicts that mostly differences between the findings of two pathologists P1 and P3 are lying between 95% confidence limits of agreement (−15.2499, 8.9971). An insignificant systematic bias was found between these two pathologists’ findings, because most of the points are clustered around the mean difference line without any clear pattern.

**Figure 8:**
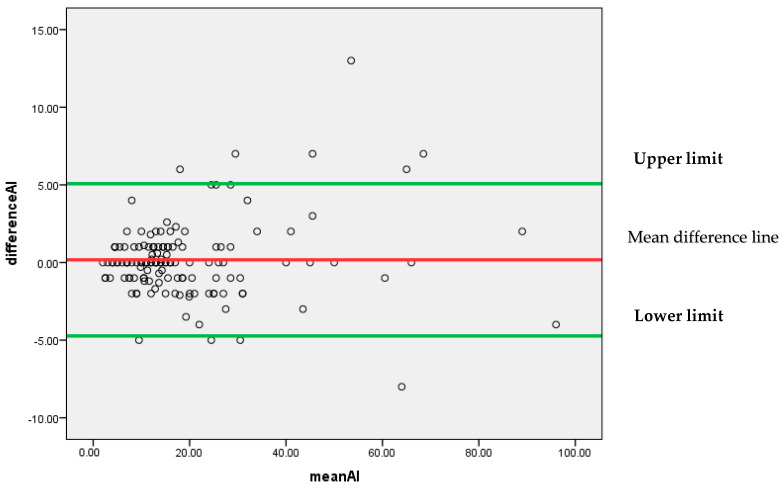
Bland–Altman plot for P1 and AI.

Figure 8 represents that most of the differences are scattered around the mean difference line without any particular pattern. All the differences between the findings of pathologist P1 and AI for the Ki-67 score lie between 95% confidence limits of agreement (−4.7302, 5.0716) with an insignificant systematic bias.

As a whole, it can be concluded by Bland–Altman analysis that the 95% confidence limit of agreement is smallest for (P1 and AI) as compared to (P1 and P2) and (P1 and P3). It indicates that AI findings can be considered reliable as an alternative to a pathologist.

## 4. Discussion

Through this study, we attempted to observe the agreement of Ki-67 scoring among three histopathologists. Comparison was also made between the outcomes of the novel DeepLiif deep learning model for Ki-67 assessment with the conventional manual Ki-67 scoring method.

The findings of Ki-67 scoring by P1 very strongly correlated with scoring by P2, P3, and AI. By comparing both methods, we were able to achieve a Cronbach’s alpha of 0.985 indicating an excellent internal consistency and an intra-class correlation coefficient (ICC) = 0.930 at 95% confidence interval (Figure 4). These findings depict outstanding reliability when comparing our results with a survey by IKWG which investigated 10 pieces of AI software as well as approximately seven scanners and observed an ICC = 0.83 at 95% CI. Another study assessed Ki 67 scoring at eight different sites utilizing one scanner and achieving an ICC of 0.89, which outperformed the pathologist-based scoring methodology at an ICC = 0.87 [5,15]. This demonstrates that the DeepLiif novel algorithm provides a statistically better outcome. Another working group on ki-67 quantification experimented with Qupath a free open-source accessible tool, and observed an ICC in the range of 0.9–0.95, which in comparison to the current algorithm gives either a lower or equivalent yield [15]. DeepLIIF has been extensively tested across multiple benchmark datasets from scanners/microscopes from different labs. It was used out-of-the-box on our microscope snapshot images which had significantly low quality due to microscope limitations than the images reported in their *Nature Machine Intelligence* paper. This shows the generalizability of the DeepLIIF approach/model [12].

Improvement in the Ki 67 scoring protocol by both manual and automated methods require standardization of inter-laboratory protocols at different levels and controlling variability among various laboratories in the preanalytical phase including type of specimen, fixation, and staining methodology. Similarly, at the interpretation level, selection of ROI, type of scanner used, quality of digital imaging, and pathologists’ experience all contribute to the outcome [16].

In the current study, we took into account the tumour hotspots for counting the Ki-67 positive cells. Calculation of a Ki-67 score by pathologist 1 was considered as the gold standard. We compared it with the sensitivity, specificity, positive predictive value, negative predictive value, as well as accuracy of the scoring performed by pathologists 2, 3, and AI, respectively (Figure 5). However, amongst them, the highest values were achieved with AI diagnosis (TABLE 4), including sensitivity = 94%, specificity = 97.5%, PPV = 98.95%, NPV = 86.67%, and diagnostic accuracy as 95%, respectively.

The current DeepLiif software of Ki67 scoring yielded an AUC = 0.993 which is an indicator of an excellent prediction model for both low and high Ki-67 values. In addition, this value is significantly higher in comparison to the values obtained by the three observers involved in the current study (Table 3). Stålhammar et al. [17], similarly, performed the digital analysis of Ki-67 in the hotspots of breast cancer counting 200 tumour cells under 40X objective. They obtained an AUC = 0.734, sensitivity = 81.5%, and specificity = 65.6%. Our results were significantly better in comparison to their study. Results reported by Stålhammar et al. [17] also are in agreement with our findings that application of digital imaging methodology biomarkers like Ki-67 can definitely boost reproducibility [17,18]. The significance of considering hotspots for biomarker evaluation lies in the fact that they exhibit intra tumour heterogeneity, are physiologically active, are good candidates for prognosis, and possess the greatest metastatic potential [19,20].

The good inter-observer agreement indicated by the Kappa score ranging between 0.660 and 0.736 showed marked improvement with a kappa score of 0.887 using AI ki-67 scoring methods, indicating very good agreement between the manual and DeepLiif AI algorithm (Table 2). In comparison to our findings, Ekholm et al. showed a kappa value between 0.83 and 0.88 of inter-observer agreement. Their work involved three different pathologists for manual scoring, similar to our study design. However, the number of cells counted in the hotspots also contributed to the difference of the outcome [21].

Various studies have agreed that straightforward guidelines need to be developed for Ki-67 biomarker quantification in breast cancer tissue by taking into consideration the pre-analytical as well as the analytical phases of the laboratory procedures [5,17,22]. Furthermore, it is now an established fact that AI-based digital methods can help mitigate the pathologist’s workload in dealing with repetitive complex tasks and their precious time can be utilized for complex cases and important decision making. Experimental AI-based studies require validation for their optimal utilization in clinical practice [23,24,25,26,27].

## 5. Conclusions

The potentials of digital pathology are hidden in the use of deep learning-based AI technologies, to produce clinically useful intuitions from large number of digitized slides with minimal user involvement. Despite all these potentials and benefits, the digital pathology revolution is not benefiting pathologists in low- and high-resource settings similarly. Most of the focus of the commercially available deep learning-based computational pathology vendors has been on the high-resource settings with expensive whole-slide image (WSI) scanners. Little attention has been paid to low-resource settings where decreasing numbers of trained pathologists are tasked with even larger caseloads and only have access to a conventional microscope and a connected digital camera to create digital images for AI analysis. In this work, we clinically validated an open-access pathologist-assisted framework for immunohistochemistry quantification against multi-pathologist manual interpretation/annotations in both low-resource settings. This was our first study in which we validated open-source software on digital images. The results were significant and we are planning more projects on larger cohorts in future.

## Figures and Tables

**Table 1:** Descriptive statistics for P1, P2, P3, and AI.

Variables	Minimum	Maximum	Median	25th Percentile (Q_1_)	75th Percentile (Q_3_)
P1	2	94	15	10	24
P2	1	100	15	10	22
P3	2	100	20	10	25
AI	2	98	14.15	10	25

**Table 2:** Measurement of agreement.

		P1		Total	Kappa	Standard Error	Strength of Agreement
		≤20	>20				
P2	≤20	92	11	103	0.660	0.071	Good
	>20	8	29	37			
	Total	100	40	140			
P3	≤20	88	4	92	0.736	0.061	Good
	>20	12	36	48			
	Total	100	40	140			
AI	≤20	94	1	95	0.882	0.043	Very Good
	>20	6	39	45			
	Total	100	40	140			

**Table 3:** Findings Evaluated by ROC.

Test Variables	AUC	95% CI	TEST QUALITY	CUT OFF VALUE	SENSITIVITY (TPR)	1–SPECIFICITY (FPR)
P2	0.940	0.904–0.976	Excellent	16.5	0.925	0.200
P3	0.934	0.893–0.975	Excellent	24.5	0.900	0.110
AI	0.993	0.980–1.000	Excellent	21.5	0.975	0.020

**Table 4:** Sensitivity, specificity, PPV, NPV and diagnostic Accuracy of P2, P3, and AI by taking P1 as gold standard (by Using Ki67 cut-off score as 20).

	Sensitivity	Specificity	PPV	NPV	DIAGNOSTIC ACCURACY
P2	92%	72.5%	89.32%	78.38%	86.43%
P3	88%	90%	95.65%	75%	88.57%
AI	94%	97.5%	98.95%	86.67%	95%

## Data Availability

This paper discloses a public dataset that can be accessed via email to the corresponding authors.
